# Pericyte Dysfunction Contributes to Vascular Cognitive Impairment Induced by Chronic Cerebral Hypoperfusion in Rats

**DOI:** 10.14336/AD.2023.0821-1

**Published:** 2024-05-07

**Authors:** Siyang Lin, Benjamin Landon, Hongxia Zhang, Kunlin Jin

**Affiliations:** ^1^Department of Pharmacology and Neuroscience, University of North Texas Health Science Center, Fort Worth, TX 76107, USA.; ^2^Department of Neurological Surgery, University of California, San Francisco, CA 94158, USA.

**Keywords:** white matter lesions, vascular cognitive impairment, chronic cerebral hypoperfusion, pericytes, CGS21680, 2VO

## Abstract

Vascular cognitive impairment (VCI) encompasses cognitive disorders associated with cerebrovascular disease, often manifesting as white matter lesions (WMLs), irrespective of precise triggers. The integrity of white matter is essential for neural communication and cognitive function maintenance. Persistent cerebral hypoperfusion-induced WMLs are now acknowledged as a key driver of VCI and dementia, though their exact formation mechanism remains unclear. Recent studies link pericyte dysfunction to diverse brain disorders like Alzheimer disease. However, the exact pathological connection between pericyte dysfunction and cognitive impairment in VCI remains unexplored. In this study, we aimed to examine whether pericyte dysfunction could impact WMLs and cognitive impairment in a rat VCI model. Using a rat model of chronic cerebral hypoperfusion-induced VCI through two-vessel occlusion (2VO), we verified that 2VO induced both WMLs and cognitive impairment. Notably, the number of pericytes in the brain was significantly altered after 2VO. Furthermore, we observed significantly increased capillary constrictions at pericyte bodies in the brains of 2VO-induced rats compared to sham-operated rats, accompanied by reduced cerebral blood flow (CBF). To tackle this issue, we administered CGS21680, a specific adenosine A2A subtype receptor agonist, intranasally twice a day for 7 days. We found that rats treated with CGS21680 exhibited a significant increase in CBF at 7 and 14 days after 2VO, compared to the vehicle group. Moreover, capillary lumens beneath pericytes also increased after the CGS21680 treatment. Importantly, the treatment led to substantial improvements in WMLs and cognitive impairment compared to the vehicle group. Our findings suggest a critical role of pericyte dysfunction in WMLs and cognitive impairment within the rat VCI model. This insight contributes to our understanding of pathogenesis and offers prospects for targeted intervention in VCI.

## INTRODUCTION

Vascular cognitive impairment (VCI) refers to various forms of cognitive disorder, from mild cognitive disturbances to full-blown dementia, caused by cerebrovascular disease (CVD) [1, 2]. VCI ranks as the second most prevalent type of dementia only after Alzheimer’s disease (AD), comprising about 15-30% of dementia cases [3, 4]. While a widely accepted pathological definition of VCI is currently lacking, diffuse white matter lesions (WMLs) are recognized as a significant pathological feature of vascular dementia. White matter accounts for approximately half of the brain volume, consisting of tracts that traverse the entire brain [5], and the integrity of white matter is critical in regulating efficient neuronal communication and maintaining cognitive function. Therefore, the disruption of white matter tracts can result in impaired communication between different brain regions and contribute to cognitive impairment [6]. Although several mechanisms have been reported to have roles in VCI, CCH-induced WMLs have been proposed as the central underlying cause. CCH is a chronic condition characterized by a long-term reduction in cerebral blood flow (CBF) due to various cerebral vascular and circulatory disorders [7]. Previous studies have documented that VCI often exhibits a higher prevalence and greater severity of WMLs, and significantly lower global cerebral blood flow compared to age-matched controls [8, 9], suggesting the link between WMLs and cognitive impairment.

The contractile capability of specific cells, such as smooth muscle cells, plays a crucial role in maintaining proper CBF. Recently, pericytes are gaining increasing attention as indispensable elements of micro-vessels, playing diverse and pivotal roles within the vascular system. Constituting a population of contractile cells, pericytes encase micro-vessel endothelium [10]. Particularly prominent in the central nervous system (CNS), the endothelial-to-pericyte ratio is approximately 1:1 to 3:1 [11]. This dense pericyte presence within the neurovascular unit—interposed among endothelial cells, astrocytes, microglia, and neurons—is essential for maintaining the integrity of the blood-brain barrier (BBB) [12, 13]. Beyond their role in BBB maintenance, CNS pericytes undertake an array of functions. These include bolstering angiogenesis and stabilizing blood vessels [14], aiding brain waste clearance [15], influencing neuroinflammation and stem cell activity [16-18]. Notably, cerebral pericytes possess distinctive contractile properties that enable them to finely modulate blood distribution through dynamic control of capillary branch diameters [19]. Previous studies have demonstrated a connection between pericyte dysfunction and several vascular disorders. For instance, after ischemic stroke, pericytes located on capillaries are observed to remain contracted even after reopening the occluded artery (recanalization). This contraction subsequently causes microvascular dysfunction and contributes to development of the “no-reflow” phenomenon [20, 21]. Moreover, pericyte dysfunction has been implicated in other conditions such as AD [22] and multiple sclerosis [23]. However, at present, there exists limited evidence establishing a link between pericyte dysfunction and VCI. Adenosine receptors (ARs) are a class of G protein-coupled receptors that are widely distributed throughout the body and highly expressed in pericyte. One of their main functions is to promote vasodilation [24]. Upon binding to specific ligands, a cascade of downstream signaling events is initiated, including the activation of the cAMP/PKA pathway and calcium signaling [25, 26]. These events collectively lead to the widening of blood vessels. An example of an adenosine receptor agonist is CGS21680, a representative adenosine A2A receptor agonist. Studies have illustrated its beneficial effects in conditions such as myocardial infarction and stroke [27, 28].

In the present study, we investigated how pericyte dysfunction affects WMLs and cognitive impairment in a rat VCI model. We induced VCI in rats through CCH and found that pericyte numbers changed, capillary constrictions increased, and CBF decreased. Administering the adenosine A2A receptor agonist CGS21680 improved CBF, leading to significant enhancements in WMLs and cognitive impairment. This highlights pericyte dysfunction's critical role in VCI-related issues.

## MATERIALS AND METHODS

### Animals

Sprague Dawley male rats (2 months old; weighing between 250-300g) were obtained from Charles River Laboratories (Wilmington, MA, USA). The rats were housed in pairs in separate ventilated cages, maintained under controlled conditions of a 12-hour light/12-hour dark cycle, and kept at a temperature range of 18 to 22 °C. Food and water were provided ad libitum. All animal procedures were approved by the Institutional Animal Care and Use Committee (IACUC) of the University of North Texas Health Science Center and conducted according to the National Institutes of Health (NIH) Guide for the Care and Use of Laboratory Animals.

### Animal surgery

The rats underwent two-vessel occlusion (2VO) surgery, also known as permanent bilateral common carotid artery occlusion (BCCAO), as per previously described procedures [29]. Initially, the rats were anesthetized with 5% isoflurane in 70% N_2_O and 30% O_2_, and then maintained under 3% isoflurane. They were positioned in the supine position, and a mid-neck incision was made after routine skin preparation. Careful separation of the bilateral common carotid arteries was performed, followed by permanent double-ligation using 4-0 silk sutures. Throughout the surgery, rectal temperature was measured and maintained at 37 ± 0.2°C using a heating blanket (Harvard Apparatus, Cat # 507220F). In the case of sham animals, bilateral common carotid artery exposure was conducted without artery occlusion.

### Intranasal administration

The CGS21680 powder (Tocris, Cat. #1063) was dissolved in a 2% dimethyl sulfoxide solution (DMSO) from Millipore Sigma (Cat. #472301) at a concentration of 100 μg/kg. The 2% DMSO solution served as the vehicle. To administer the solution, the rats were manually restrained to limit head movement and maintain a vertical position. Using a pipette, a small drop of the solution was placed at the nasal opening, allowing the rats to inhale the droplet. A total of 48 μl was administered by delivering 4 drops of 12 μl each, with the drops alternated between the nostrils. There was a 5-minute interval between each drop. The administration was repeated twice per day and continued for one week. During these procedures, the nostrils were always kept open. As a control, the Sprague Dawley (SD) rats were treated with 2% DMSO.

### Morris Water Maze (MWM) test

Both the sham-operated and 2VO rats underwent the Morris water maze test on day 14 to evaluate their cognitive abilities. The tests were meticulously monitored and quantitatively analyzed using ANY-maze software (Stoelting Co, Wood Dale, IL, USA), following established protocols as described in previous studies [30]. The experiments were conducted in a quiet room. A circular tank filled with warm water, maintained at a temperature of approximately 23-25 °C, was utilized for the test. Inside the tank, a hidden circular platform remained fixed 1.5cm below the water surface throughout the duration of the tests. To conceal the platform, powdered tempera blue paint was added to the water, making it indistinguishable. The tests were carried out over five consecutive days, with each rat undergoing three trials per day. Each trial had a maximum duration of 90 seconds and employed different start positions. The rats were positioned in designated start positions within the maze, facing the sidewalls of the swimming pool. If a rat failed to reach the platform within the allotted 90 seconds, it was gently guided to the platform. Once the rat reached the submerged platform, it remained there for 10 seconds before the commencement of the subsequent trial. The time taken by each rat to reach the escape platform from the start position was recorded as the escape latency. Additionally, the swimming distance, speed, and path were also recorded during the tests.

### Real-time cerebral imaging of blood perfusion

Cerebral blood perfusion (CBP) was assessed using the PeriCam PSI System (Perimed Medical Science, Las Vegas, NV, USA), which utilizes laser speckle contrast analysis (LASCA) technology. The rats were anesthetized with isoflurane and positioned in a prone position on a heated pad to maintain their body temperature. A midline incision was made on the scalp, and careful cleaning of the skull was performed to control bleeding. The laser, directed through the skull, captured the movement of red blood cells, producing speckle contrast images. The PeriCam PSI system was employed to scan the region of interest in real-time for a duration of 30 seconds, recording the dynamic and spatial distribution of blood perfusion. Cerebral blood perfusion was quantified in perfusion units (PU), arbitrary units, using PimSoft v.1.5 software (Perimed, Sweden).

### Western Blot

The rats were anesthetized using isoflurane inhalation and then underwent cardiac perfusion with ice-cold PBS. The brain regions, including the corpus callosum and striatum, were immediately dissected and lysed in RIPA buffer (containing 1% Triton-X100, 0.5% sodium deoxycholate, 0.1% SDS). The protein concentration was determined using the Quick Start Bradford protein assay (Bio-Rad, Cat# 5000201). SDS-PAGE gels with 8-10% concentration were used to separate the proteins, which were subsequently transferred to a polyvinylidene difluoride (PVDF) membrane (Merck Millipore, Cat# IPVH00010). The membranes were blocked with a solution of 5% non-fat milk and 0.1% Tween 20 for 1 hour at room temperature. Following this, the membranes were incubated overnight at 4°C with specific primary antibodies, including anti-MBP (1:1000, Novus Biologicals, Cat #50035), anti-Neurofilament H (1:200, BioLegend, Cat #801701), and anti-β-actin (1:1000, Santa Cruz Technology, Cat #47778). The membranes were then incubated with secondary antibodies labeled with horseradish peroxidase, such as goat anti-mouse and anti-chicken IgG (1:1000, Abcam, Cat #ab 6877 and 205719). Protein signals were detected using Pierce enhanced chemiluminescence (ECL) substrate (ThermoFisher Scientific, Cat# 32106), and the data were captured using the ChemiDoc Imaging System (Bio-Rad, USA). The density of the target protein bands was quantified using FIJI-ImageJ software (NIH, USA), with β-actin used for normalization.

### Immunohistochemistry

Coronal brain sections (7-µm-thick) were obtained after fixation and paraffin embedding. The sections were then rehydrated and boiled in Citrate buffer (pH 6.0, Sigma-Aldrich, Cat #71402) in a microwave for 10 minutes. After incubation in a blocking buffer (containing 5% donkey serum, 3% bovine serum albumin, and 0.1% Triton X) at room temperature for 1 hour, the sections were incubated overnight at 4°C with the primary antibodies. The primary antibodies used included anti-MBP (1:1000, Novus Biologicals, Cat #50035), anti-Neurofilament H (1:200, BioLegend, Cat #801701), anti-PDGFRβ(1:100, Abcam, Cat #ab32570), anti-Lectin (1:200, Vector Laboratories, Cat #DL-1174), anti-αSMA (1:200, Abcam, Cat #ab7817), anti-Iba1 (1:1000, WAKO, Cat#011-27991), anti-NeuN (1:100, EMD Millipore, Cat #MAB377), anti-GFAP (1:1000, ThermoFisher Scientific, Cat #PA1-10004), anti-Cleaved Caspase-3 (1:200, Cell Signaling, Cat #9669), and anti-MCM2 (1:200, ThermoFisher Scientific, Cat #MA5-15895). After washing with PBS, the sections were incubated in the dark at room temperature for 1 hour with the appropriate secondary antibodies. The secondary antibodies used were Alexa Fluor 488-, 594-, or 647-conjugated donkey anti-rabbit, anti-mouse, anti-chicken, or anti-goat IgG (1:1000, ThermoFisher Scientific), and biotinylated anti-rabbit IgG (1:200, Vector Laboratories, Cat #BA-1100). Nuclei were counterstained with DAPI (4’,6-diamidine-2-phenylindole dihydrochloride, ThermoFisher Scientific, Cat #S36973). Fluorescent labeled images were captured using an Olympus Fluoview FV1200 confocal microscope (Olympus Life Science, Tokyo, Japan), while biotin-labeled images were taken using a Nikon Eclipse TI microscope (Nikon corporation, Tokyo, Japan). The fluorescence intensity of each region of interest (ROI) was measured using FIJI-ImageJ software (NIH, USA).

### Visualization of Trapped Erythrocytes

To visualize the trapped erythrocytes in the capillaries, the brain sections were treated with sodium borohydride (NaBH4), which allows for the detection of the fluorescence emitted by the trapped erythrocytes. The sections were obtained from rat brains that had undergone perfusion and were initially immersed in xylene to remove the embedding medium. Subsequently, the sections were rehydrated using a series of alcohol solutions with decreasing concentrations, followed by rinsing with PBS to remove any residual chemicals. Next, the sections were treated with a solution containing 0.2% NaBH4 (Sigma-Aldrich, Cat #213462) in PBS for 30 minutes. This treatment enhances the fluorescence of the trapped erythrocytes, allowing for their visualization. After the treatment, the sections were rinsed with PBS for 10 minutes to remove any remaining NaBH4. The resulting fluorescent images of the trapped erythrocytes in the treated sections were captured using an Olympus Fluoview FV1200 confocal microscope, enabling their visualization and analysis.

### Histopathology

Luxol fast blue (LFB) staining was used to evaluate changes in the structural integrity of myelin. Following deparaffinization and rehydration, the sections were placed into an LFB solution (containing 0.1% LFB, 95% alcohol and 10% acetic acid) (Sigma-Aldrich, Cat #S3382) overnight at 60°C and then cooled to ambient temperature. Excessive staining was removed by distilled water rinses for 1 min each. After washing the sections with 95% ethyl alcohol and distilled water, white matter was differentiated from gray matter in 0.5% lithium carbonate solution (Sigma-Aldrich,Cat #255823) for 30 seconds, followed by 70% ethanol for 30 s, then rinsed in ddH2O. Repeat the differentiation process until the unmyelinated tissue appeared white under the microscope. Images were captured using Nikon Eclipse TI microscope.

### Statistical analysis

All the results are presented as mean ± standard error of mean (SEM). Statistical analysis was performed using GraphPad Prism 8 (GraphPad Software, San Diego, CA, USA). The sample size was determined using power analysis with G*Power software (Heinrich-Heine-Universität Düsseldorf, Düsseldorf, Germany; http://www.gpower.hhu.de/), based on a statistical test for the difference between two independent means, with a significance level (α) of 0.05 and a power of 80%, assuming an effect size of 1.2. The data normality was determined using Shapiro-Wilks test. For normally distributed populations of data points, Student t-test (2 groups) or a one-way ANOVA followed by Tukey post-hoc test (>2 groups) were used. For data that failed the normality test, the Mann-Whitney test was used. For the data from Morris water maze test, repeated measure ANOVA was used followed by Bonferroni post hoc test. A p-value of less than 0.05 was considered statistically significant.

## RESULTS

### Morphological characteristics of pericytes in the brain

**Figure 1.**
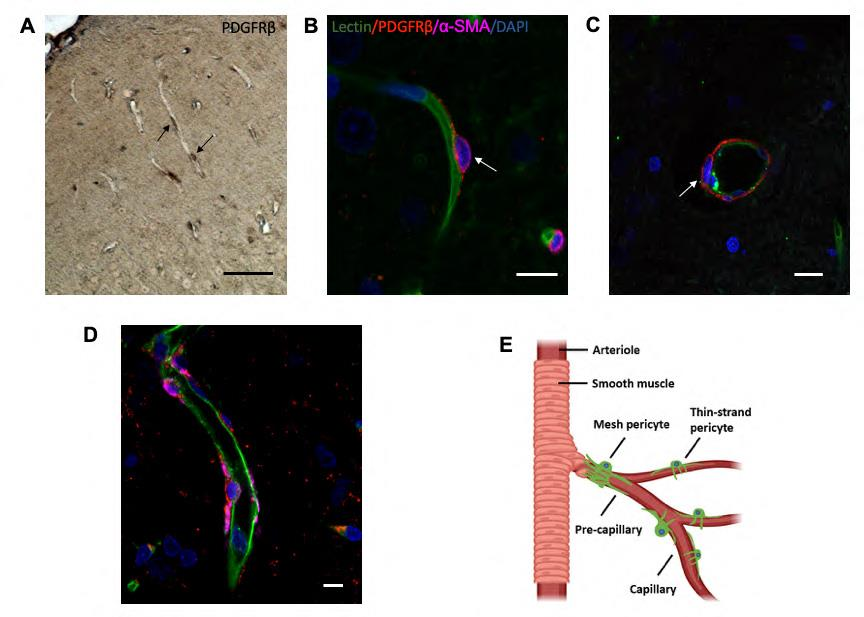
**Morphological Characteristics of Pericytes Associated with Cerebral Capillaries**. (**A**) DAB staining highlights pericytes along the capillary network. Pericytes are identified as 'bumps' or 'crescent'-shaped cells, indicated by black arrows. The location of pericyte cell bodies along the capillary wall is shown. Scale bar = 50 μm. (**B-D**) Brain sections stained with lectin (green) and immunostained with PDGFRβ (red) and αSMCs (far red). DAPI (blue) was used for nuclear counterstaining. Fluorescent images show pericytes located on capillaries and pre-capillaries. The distinctive 'bump-on-a-log' morphology of pericytes is observed on the outer surface of capillaries (B), as indicated by white arrows. In pre-capillaries, pericytes express αSMA (D) and form an encircling pattern around the lumen (C). Scale bar = 10 μm. (**E**) A schematic diagram illustrates the arrangement of mesh pericytes and thin-strand pericytes along capillaries and pre-capillaries.

One of the main challenges in studying pericytes is the lack of a universally accepted marker that specifically distinguishes them from other types of mural cells. Pericytes are typically characterized by their unique morphology, which includes an oval-shaped cell body and long processes that wrap around blood vessels. The protein Platelet-Derived Growth Factor Receptor β (PDGFRβ) has conventionally been used as a marker for identifying pericytes. In our study, we identified pericytes as distinct nodule-like "bumps" or "crescent" shaped cells that exhibited immunostaining with PDGFRβ and were closely associated with the basement membrane of microvessels. These pericytes were observed at irregular intervals within the microvascular network of both the cerebral cortex and white matter. They were particularly prominent along the capillary wall (Fig. 1A), especially in thicker tissue sections. Anatomically, brain pericytes are located directly on small blood vessels, including capillaries, pre-capillary arterioles, and post-capillary venules (Fig. 1E). It has long been recognized that the structure, morphology, and distribution of pericytes vary along the arterial-venous axis. In the mid-capillaries, pericytes known as "thin-strand pericytes" exhibit elongated cell bodies and extend thin strands or helical processes that run parallel to the vessel lumen (Fig. 1B). A subpopulation of pericytes, referred to as "mesh pericytes", was found at the pre-capillary arterioles (Fig. 1C, D). These pericytes are located at the transition between arterioles and capillaries and share some characteristics with smooth muscle cells (SMCs). They display an elongated shape, encircling the entire vessel lumen, and also possess protruding oval cell bodies similar to mid-capillary pericytes.

### Increased pericyte numbers in the brain following chronic cerebral hypoperfusion

**Figure 2.**
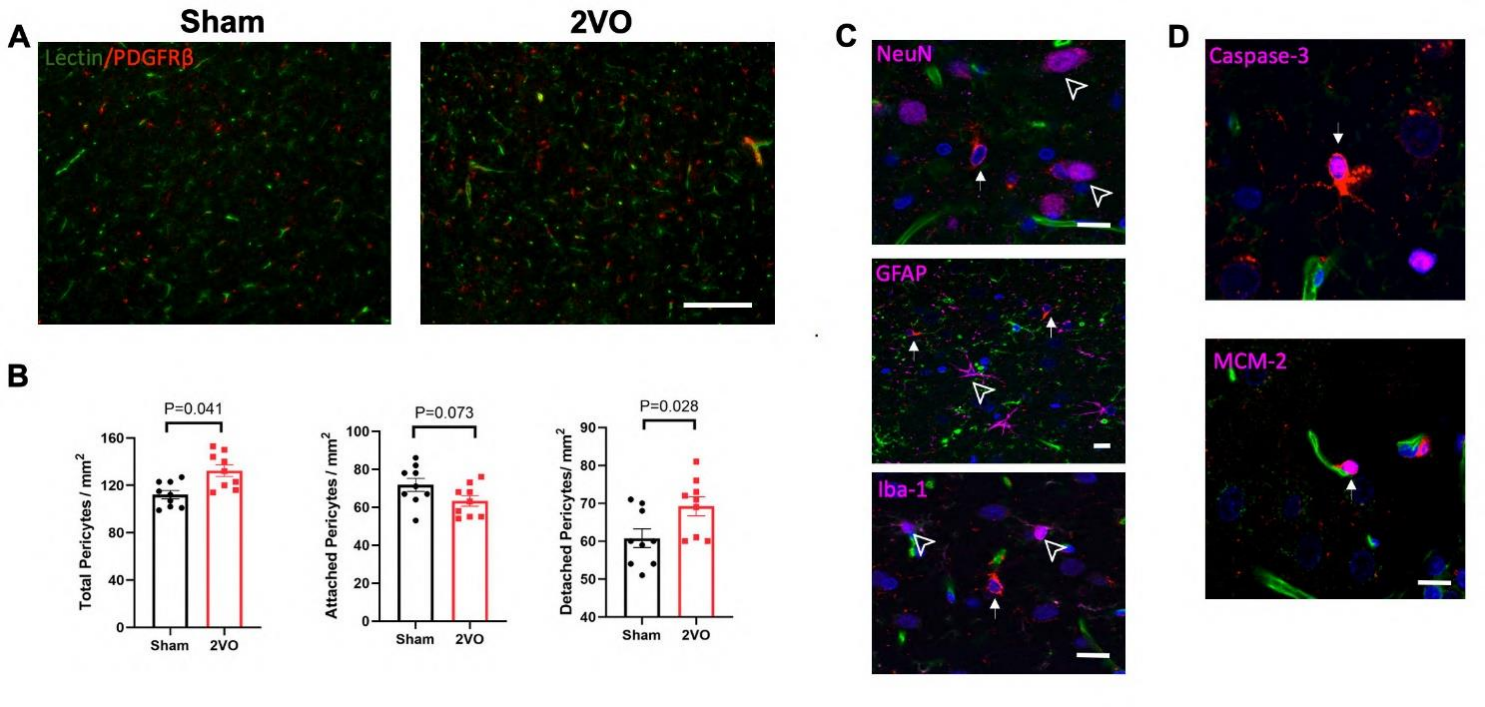
**Changes in Pericyte Numbers following Chronic Cerebral Hypoperfusion**. (**A**) Representative images display both attached and detached pericytes in the sham and 2VO groups. The scale bar represents 100 μm. (**B**) Quantitative analysis presents the total number of pericytes (left panel), attached pericytes (middle panel), and detached pericytes (right panel) in the sham and 2VO groups. The sample size was N=9 per group. The P values were assessed by unpaired Student’s t test. (**C**) Representative confocal images demonstrate detached pericytes co-stained with NeuN (top panel), GFAP (middle panel), and Iba-1 (bottom panel). Detached pericytes are indicated by white arrows, while neurons (top panel), astrocytes (middle panel), and microglia (bottom panel) are indicated by black arrowheads. The scale bar represents 20 μm. (**D**) Representative images display detached pericytes co-stained with Caspase-3 (top panel) and attached pericytes with MCM-2 (bottom panel). The scale bar represents 20 μm.

Following chronic cerebral hypoperfusion, rats were examined for changes in pericyte numbers and their functional status. Pericytes were labeled using PDGFRβ antibody, while capillaries were visualized using fluorescently labeled lectin. After 14 days of two vessel occlusions (2VO), a significant increase in the total number of pericytes was observed in the 2VO group compared to the sham group. Notably, detached PDGFRβ-positive pericytes located away from capillaries were also observed (Fig. 2A). The increase was primarily attributed to the elevated number of detached pericytes, whereas the number of attached pericytes showed no statistical difference between the two groups (Fig. 2B). These findings highlight the impact of chronic cerebral hypoperfusion on pericyte populations, with a particular emphasis on the presence of detached pericytes.

To investigate the phenotypes of the detached pericytes, immunofluorescence staining was performed using antibodies against NeuN (for neurons), GFAP (for astrocytes), and Iba-1 (for microglia). As depicted in Figure 2C, the detached PDGFRβ+ pericytes did not exhibit colocalization with neurons or glial cells, suggesting that they are distinct from these cell types. To further characterize the detached pericytes, we co-stained them with cleaved Caspase-3, an enzyme associated with apoptosis, and MCM-2, a marker for cell replication. Interestingly, the majority of the detached pericytes (approximately 60%) displayed signs of apoptosis, indicating their involvement in apoptotic processes. In contrast, a subset of attached pericytes (approximately 30%) exhibited the expression of MCM-2, suggesting their potential as newly generated cells that could contribute to angiogenesis. These findings provide insights into the diverse phenotypes and functional states of detached and attached pericytes in the context of chronic cerebral hypoperfusion.

### Decreased microcirculation and altered capillary morphology following chronic cerebral hypoperfusion

To assess the impact of chronic cerebral hypoperfusion on microcirculation, we employed laser speckle contrast analysis (LASCA) technology. Initially, baseline measurements of cerebral blood flow (CBF) were recorded for 30 seconds under anesthesia. Following this, another 30 seconds CBF measurement was conducted immediately after inducing two vessel occlusions (2VO).

**Figure 3.**
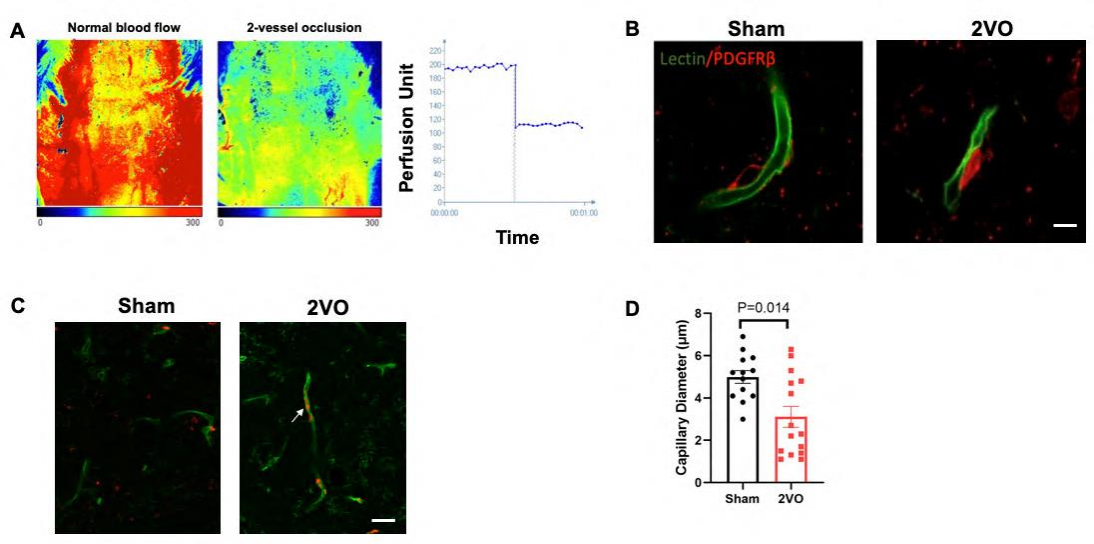
**Decreased microcirculation following chronic cerebral hypoperfusion**. (**A**) Laser speckle contrast images showing the pattern of normal blood flow (left panel) and reduced blood flow immediately after 2VO (middle panel). Graph of perfusion units demonstrating the change in perfusion over time before and after 2VO (right panel). (**B**) Representative fluorescent images displaying a normal capillary lumen (left panel) and a constricted lumen observed after 2VO (right panel). Scale bar = 10 μm. (**C**) Representative fluorescent images illustrating the fluorescence of hemoglobin after NaBH4 treatment. Numerous trapped erythrocytes can be observed in capillaries after 2VO (right panel). White arrows indicate entrapped erythrocytes around nodal constrictions. Scale bar = 30 μm. (**D**) Quantitative analysis of capillary diameter in the sham (N=13) and 2VO (N=15) groups, demonstrating a significant decrease in capillary diameter in the 2VO group compared to the sham group. The P value was assessed by Mann-Whitney test.

Representative images of cerebral blood perfusion were captured, illustrating the levels of CBF before and after 2VO (Fig. 3A, left and middle panel). The CBF in the cerebral hemispheres immediately after 2VO was found to be approximately 52% compared to baseline (Fig. 3A, right panel), indicating a successful induction of the 2VO model.

Furthermore, to examine the impact of chronic cerebral hypoperfusion on pericyte function, we stained the pericytes with PDGFRβ antibody and the capillaries with Lectin. We observed a slight constriction of capillary lumens due to the contractile ability of pericytes. Interestingly, after 14 days of chronic cerebral hypoperfusion, a significant reduction in capillary diameter was observed compared to the sham group (Fig. 3B). Quantitative analysis revealed that the capillary diameter in the sham group measured 4.99 ± 0.30 µm, whereas it was reduced to 3.10 ± 0.49 µm in the 2VO group (Fig. 3D).

To visualize the occluded capillaries more directly, tissue samples were treated with sodium borohydride (NaBH4). NaBH4 acts as a strong reducing agent, causing trapped erythrocytes to become highly fluorescent by releasing iron from its quenching position within the hemoglobin's porphyrin ring [31]. As a result, numerous trapped erythrocytes were observed in capillaries after 2VO, while the sham group exhibited only background autofluorescence (Fig. 3C).

### Reversing pericyte contraction pharmacologically improves cerebral circulation following 2VO

To investigate the potential of inhibiting pericyte contraction for improving cerebral reperfusion after chronic cerebral hypoperfusion, rats were treated with either CGS21680 or a vehicle following the induction of 2VO. CGS21680 is a selective adenosine A2A receptor agonist. After the treatment, changes in cerebral blood flow (CBF) were monitored using laser speckle contrast analysis (LASCA) over a period of 2 weeks.

**Figure 4.**
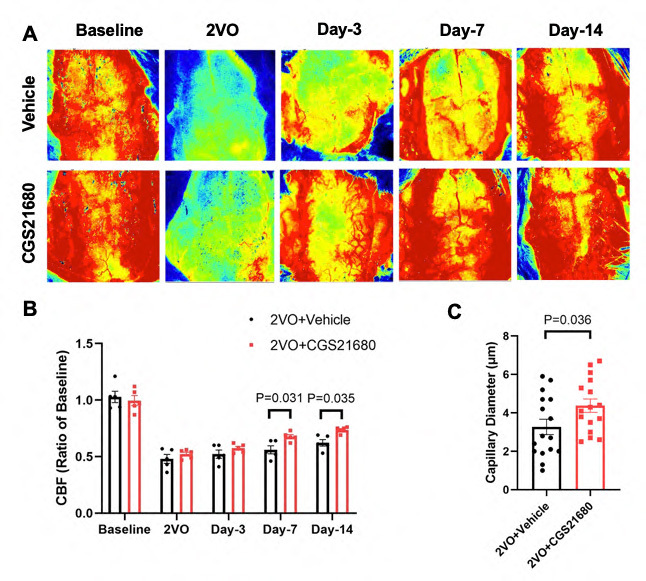
**Inhibiting pericyte contraction improves cerebral circulation following 2VO**. (**A**) Representative laser speckle contrast images depict the time course of cerebral blood flow changes in the vehicle group (top panel) and the treatment group (bottom panel) following 2VO. These images visually represent the alterations in blood flow over time. (**B**) Quantitative analysis of cerebral blood flow changes was performed in both the vehicle and treatment groups after 2VO over a 2-week period. The results are presented graphically, showing the percentage change in cerebral blood flow compared to baseline for each time point in the two groups. The sample size was N=5 per group. The P values were assessed by mixed-design ANOVA followed by Bonferroni’s multiple comparisons test. (**C**) Capillary diameter was quantitatively analyzed in both the vehicle and treatment groups (N=16 per group). The measurements of capillary diameter in each group are compared, providing insights into the impact of the treatment on capillary size. The P value was assessed by unpaired Student’s t test.

Figures 4A and 4B depict the time course of CBF changes in both the vehicle and CGS21680 treatment groups following 2VO. Initially, a sharp decrease in CBF was observed immediately after 2VO, followed by a gradual recovery over time. After 3 days of recovery, CBF was reduced by 48% in the vehicle group and 43% in the CGS21680 treatment group. Importantly, a significant difference in CBF between the two groups was noted on day 7 after 2VO, with the treatment group exhibiting 67% CBF compared to baseline, while the vehicle group had 56% CBF compared to baseline (P=0.031). This difference persisted at the 14-day mark, with the vehicle group showing a 38% decrease in CBF compared to baseline, whereas the treatment group demonstrated a 27% decrease (P=0.035). These findings suggest that inhibiting pericyte contraction with CGS21680 may lead to improved cerebral reperfusion following chronic cerebral hypoperfusion.

In addition to monitoring cerebral blood flow, we also examined the condition of microcirculation by comparing the capillary diameter between the vehicle and treatment groups. Quantitative analysis showed that the capillary diameter in the treatment group was 4.32 ± 0.28 µm, while in the vehicle group, it was reduced to 3.27 ± 0.40 µm. This finding suggests that treatment with CGS21680 leads to the dilation of microvessels.

Furthermore, we assessed the quantity of pericytes, including both attached and detached pericytes, in both the vehicle and treatment groups. However, no notable differences were observed between the two groups. The total number of pericytes, as well as the number of attached and detached pericytes, did not show significant variations. These results indicate that the treatment with CGS21680 did not have a substantial impact on the quantity of pericytes in the context of this study.

**Figure 5.**
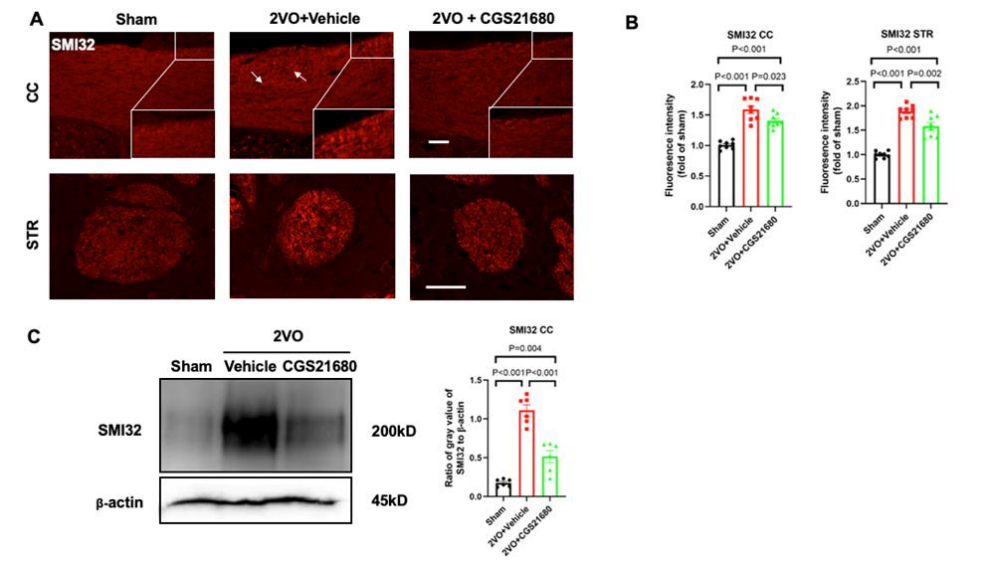
**Inhibiting pericyte contraction attenuates axonal damage**. (**A**) Representative immunofluorescence images of SMI32 (red) labeling in the corpus callosum (top panel) and striatum (bottom panel) are displayed for the sham, vehicle, and treatment groups at 14 days after 2VO. The insert shows a higher magnification view of the corpus callosum. White arrows indicate damaged axonal fibers. Scale bar = 50 μm. (**B**) Quantification of the fluorescence intensity of SMI32 in the corpus callosum and striatum of each group is calculated as the fold change over the sham group. The sample size was N=8 per group. (**C**) Western blot analysis of protein samples obtained from the corpus callosum using the SMI32 antibody (left panel). (N=6 per group) Densitometric analysis of SMI32 protein levels in each group is presented (right panel). The values in the graph represent the SMI32/β-actin ratio, with β-actin serving as a housekeeping protein. All the P values were assessed by one-way ANOVA followed by Tukey post-hoc test.

### Inhibiting pericyte contraction attenuates axonal damage

To evaluate the effect of CGS21680 treatment on axonal damage during chronic cerebral hypoperfusion (CCH), we focused on assessing the integrity of white matter, including myelin and axonal components. Specifically, we examined the corpus callosum and striatum, which are commonly studied regions in white matter research.

To detect axonal damage, we utilized the SMI32 antibody, which specifically targets non-phosphorylated neurofilaments and serves as a marker for axonal injury. In the sham group, minimal expression of SMI32 was observed, indicating intact axonal structures. However, the vehicle group displayed increased expression of SMI32, indicating axonal damage in response to CCH.

In contrast, the group treated with CGS21680 showed a decrease in the fluorescence intensity of SMI32 in both the corpus callosum and striatum, suggesting a reduction in axonal damage compared to the vehicle group (Fig. 5A, 5B). These findings were further supported by Western Blot analysis, which demonstrated decreased levels of SMI32 in the CGS21680-treated group compared to the vehicle group (Fig. 5C).

Overall, these results indicate that inhibiting pericyte contraction with CGS21680 attenuates axonal damage in the context of chronic cerebral hypoperfusion, suggesting a potential therapeutic benefit for preserving white matter integrity.

### Inhibiting pericyte contraction ameliorates myelin loss

**Figure 6.**
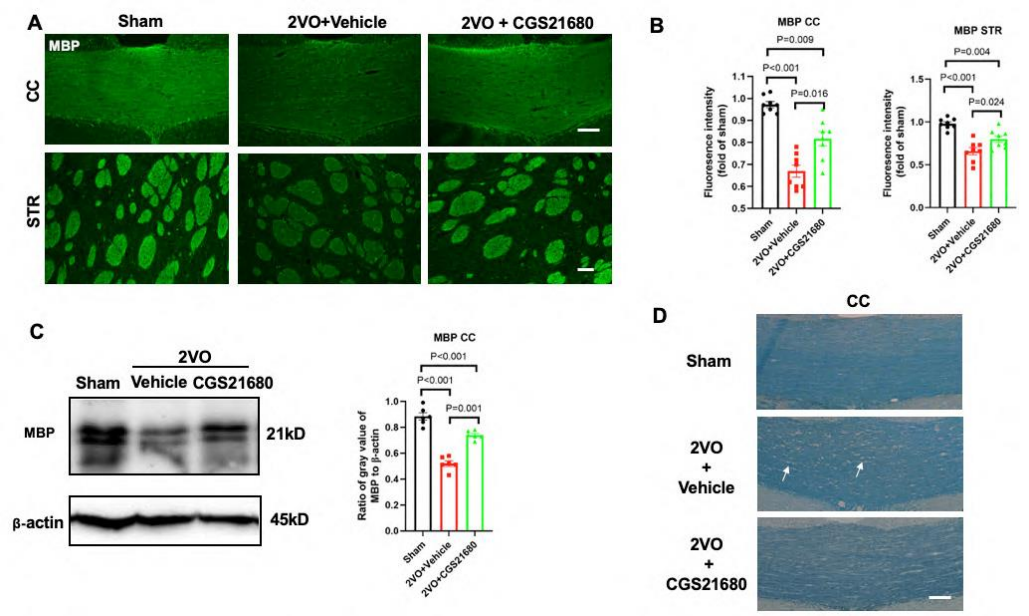
**Inhibiting pericyte contraction ameliorates myelin loss**. (**A**) Representative images of MBP (green) immunofluorescence labeling in the corpus callosum (top panel) and striatum (bottom panel) are presented for the sham, vehicle, and treatment groups at 14 days after 2VO. Scale bar = 50 μm. (**B**) Quantification of the fluorescence intensity of MBP in the corpus callosum and striatum for each group. The values are calculated as the fold changes over the sham group. The sample size was N=8 per group. (**C**) Western blot analysis of protein samples obtained from the corpus callosum using an MBP antibody (left panel) (N=6 per group). Densitometric analysis of MBP expression in each group (right panel). The values on the graph represent the ratio of MBP to β-actin, with β-actin utilized as a housekeeping protein. (**D**) Representative images of Luxol fast blue (LFB) staining in the corpus callosum. White arrows indicate the presence of vacuoles. Scale bar = 50 μm. All the P values were assessed by one-way ANOVA followed by Tukey post-hoc test.

MBP (myelin basic protein) is a marker that indicates the integrity of myelin. As expected, after 14 days of cerebral hypoperfusion, the regions of the corpus callosum and striatum showed reduced fluorescence staining of myelin and decreased expression of MBP in Western Blot analysis, compared to the sham group. In contrast, the CGS21680 treatment group exhibited enhanced fluorescence intensity of MBP and increased protein expression (Fig. 6A-C). Additionally, Luxol fast blue (LFB) staining, commonly used to assess white matter lesions by staining the lipoproteins of the myelin sheath, revealed reduced intensity in the vehicle group, accompanied by myelin damage such as vacuolization or loss of the myelin sheath. Notably, CGS21680 treatment significantly mitigated myelin damage compared to the vehicle group (Fig. 6D).

### Inhibiting pericyte contraction improves cognitive functions

**Figure 7.**
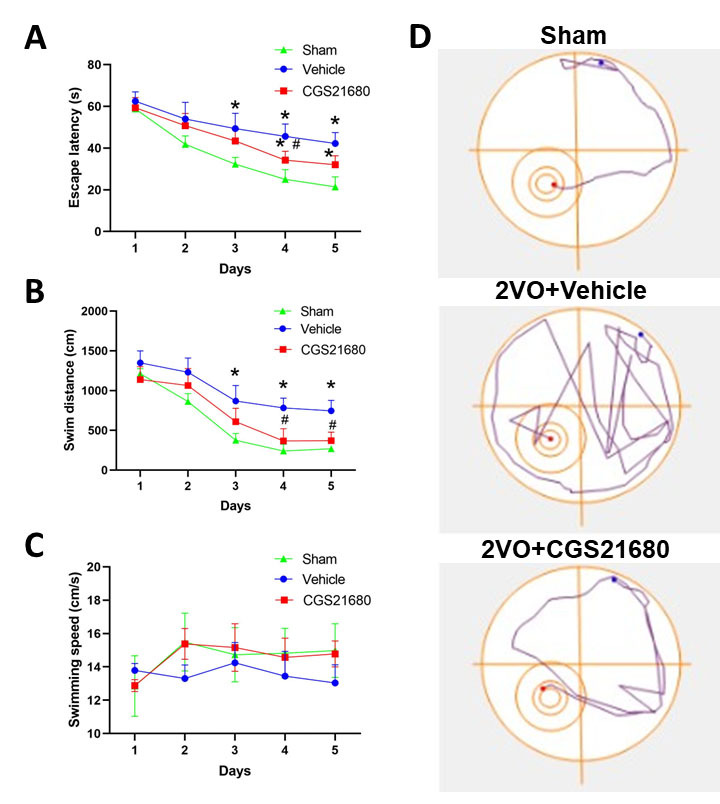
**Inhibiting pericyte contraction improves cognitive functions**. (**A**) Vehicle and CGS21680 were administered to Sham or 2VO rats for one week, followed by the performance of the Morris water maze test at 14 days post-treatment. The results show the average time taken to reach the platform (escape latency) in the Morris water maze. (**B**) The average cumulative distance covered in the Morris water maze was measured in both sham and 2VO rats treated with vehicle or CGS21680. (**C**) Mean swimming speed to the platform in the Morris water maze was assessed in both sham and 2VO rats treated with vehicle or CGS21680. (**D**) Representative swimming paths of different groups during the acquisition trial. The sample size was N=12 in sham and treatment groups, N=11 in vehicle group. P values were assessed by two-way repeated ANOVA followed by Bonferroni’s multiple comparisons test. * indicates significant difference between vehicle and sham groups while # indicates significant difference between vehicle and treatment groups. ^*^P<0.05, ^**^P<0.01, ^***^P<0.001 and ^#^P<0.05

To assess the effects of chronic cerebral hypoperfusion and CGS21680 treatment on memory and spatial learning impairments, we employed the Morris Water Maze (MWM) test. The acquisition trial of the MWM task measures learning capacity by analyzing the escape latency (time taken to reach the platform) and swimming distance to the platform. Statistical analysis using repeated-measure ANOVA demonstrated significant differences in escape latency and swimming distance among the sham, vehicle, and CGS21680 treatment groups. Post hoc tests revealed that CGS21680 treatment led to a significant decrease in escape latency on day 4 and swimming distance on day 4 and 5, compared to the vehicle group (Fig. 7A-B). However, no significant difference in swimming speed was observed among the three groups (Fig. 7C). The observed escape routes in the MWM test reflect the strategies employed by the rats to find the platform. As depicted in Figure 7D, the sham group exhibited a short and direct route, while the vehicle group displayed a more intricate and convoluted route. The CGS21680 treatment group showed improvement compared to the vehicle group, although their routes were still inferior to those of the sham group.

## DISCUSSION

In this study, our observations revealed a substantial increase in the number of both total and detached pericytes within the brain after 2VO, when compared to sham-operated rats. Moreover, a distinct elevation in capillary constrictions, particularly centered around pericyte bodies, was noted following CCH. Interestingly, our investigation demonstrated that the inhibition of pericyte constriction through the utilization of CGS21680 yielded significant improvements in WMLs and cognitive impairment, as compared to the vehicle treatment. These findings underscore the pivotal role of pericyte dysfunction in contributing to WMLs and cognitive impairment within the rat model of VCI.

As the aging population continues to grow, dementia has emerged as one of the prominent health challenges in our society. The prevalence of VCI is expected to rise significantly, with current estimates ranging from 8 to 17 million cases globally, projected to triple by 2050 [32]. While an exact pathological definition of VCI remains elusive, WMLs induced by CCH are widely acknowledged as a pivotal mechanism contributing to VCI's development.

White matter constitutes a substantial portion of the brain, housing tracts that interconnect cortical and subcortical gray matter regions, forming essential neural networks [33]. CCH precipitates a reduction in cerebral oxygen supply, which subsequently disrupts the regular function of white matter cells, notably oligodendrocytes, leading to demyelination, axonal degeneration, and the emergence of WMLs [34]. Deficits in white matter consistently emerge as a core element in the underlying pathology of VCI. Cognitive slowing stands as the primary manifestation of white matter impairment, stemming from compromised impulse transmission due to damaged myelin and, in some cases, axonal injury. This impairment also extends to executive function, memory retrieval, sustained attention, and an array of psychiatric disorders [35].

Cross-sectional studies have highlighted the correlation between VCI and decreased CBF, along with the presence of more pronounced WMLs observed on MRI in comparison to age-matched individuals without cognitive impairment [36]. In our study, we successfully established a rat model of chronic hypoperfusion through two-vessel occlusion, closely mirroring the hypoperfusive state witnessed in VCI. Consistent with prior research, we observed a significant decline in cerebral blood flow after two-vessel occlusion, reaching approximately 40-50% of the control level, which gradually recuperated over an 8-week span. Immunofluorescence and LFB staining results further substantiated the occurrence of myelin loss and axonal damage post two-vessel occlusion. Additionally, the prolonged latency and increased swimming distance exhibited by the two-vessel occlusion group during the Morris Water Maze test pointed to spatial learning deficits following the surgical procedure.

Pericytes, integral components of the vascular system, play a pivotal role in maintaining the integrity and functionality of blood vessels. Discovered in the 1870s, these cells exhibit a distinctive bump-on-a-log appearance and are typically located on the outer surface of capillaries [37]. Depending on their location relative to micro-vessels, pericytes can be classified into three types: 1) Precapillary pericytes: These are situated at the transition point between arterioles and capillaries, demonstrating a hybrid phenotype resembling both smooth muscle cells (SMCs) and pericytes. They are often referred to as "mesh pericytes" due to their interlocking mesh-like arrangement. 2) Capillary pericytes: Characterized by cell bodies that extend with delicate strands or helical primary processes aligned parallel to the long axis of mid-capillary tubes. 3) Post-capillary pericytes: These are characterized by numerous slender and branching processes and become progressively more abundant on post-capillary venules [38].

Presently, there lacks a single specific marker exclusively employed for pericyte identification. Markers commonly used for this purpose are also expressed in other brain cell types, posing challenges to discerning pericytes solely based on these markers. Additionally, these markers exhibit dynamic expression patterns in pericytes, differing during various developmental stages, pathological conditions, and even under diverse culture conditions [39]. Existing research reveals that pericytes express a range of markers, including α-SMA, smooth muscle myosin, tropomyosin, nestin, vimentin, desmin, neuron glial antigen 2 (NG2), regulator of G protein signaling 5 (RGS5), and PDGFRβ [40, 41]. Among these markers, PDGFRβ has conventionally been utilized as a marker for pericytes due to its significance in pericyte recruitment and vascular stability during development [42]. Consequently, leveraging a combination of markers or co-localization studies enhances the accuracy of pericyte identification. In our study, we employed PDGFRβ as a marker for pericytes, coupled with Lectin, an antibody that labels the basal membrane of endothelial cells. This approach allowed us to observe the distinctive morphology of pericytes, resembling "bumps" or "crescents," closely associated with the basal membrane of micro-vessels, through both DAB staining and fluorescent staining. Furthermore, we noted the presence of mesh pericytes forming an encircling pattern around the vessel lumen. Additionally, the co-localization of pericytes with antibodies against α-SMA indicated their capability to contract and modulate blood flow.

Pericytes exhibit diverse origins based on their embryonic location. CNS pericytes stem from neural crest cells, while pericytes in peripheral organs primarily originate from the mesothelium [39, 43]. These distinct embryonic origins imply separate biological roles for CNS and peripheral pericytes. In later embryonic stages and early postnatal periods, the escalation in brain pericyte population is primarily fueled by the proliferation and expansion of pre-existing pericyte pools [43, 44]. In ischemic regions, pericyte progenitors have been observed around developing blood vessels. These progenitors differentiate into pericytes, expressing TGF-β and VEGF [45]. Despite substantial progress in understanding embryonic brain pericyte origins, knowledge gaps persist regarding how pericyte replenishment and replacement occur in the adult and aging brain under normal physiological circumstances. Within the brain, pericytes have an active role in modulating capillary diameter in response to diverse stimuli, including depolarization, neurotransmitters, and neuronal activity [46, 47]. Research indicates that capillaries dilate before arterioles in response to neuronal activity, with the presence of a pericyte on the vessel correlating to capillary dilation [47, 48]. This suggests that capillary dilation isn't solely a passive outcome of arteriole dilation-induced elevated blood pressure. Instead, it represents an active relaxation response by pericytes. Our study revealed a notable reduction in capillary lumen specifically at pericyte bodies following chronic cerebral hypoperfusion, in contrast to the sham group. This suggests that pericyte contraction induced by chronic cerebral hypoperfusion results in capillary constriction. Furthermore, the application of NaBH4 indicated capillary occlusion, evident from the entrapment of erythrocytes within the lumen. As cardiac perfusion was conducted before decapitation, the presence of erythrocytes in the lumen points to an abnormal condition.

The observed normalization of CBF over time in the 2VO model suggests the presence of compensatory or adaptive processes. In these compensatory mechanisms, pericytes play a critical role in angiogenesis. When exposed to pro-angiogenic signals, pericytes respond by expressing various matrix metalloproteinases (MMPs), which degrade components of the basement membrane. This process leads to the detachment of pericytes, facilitating the release of endothelial cells [49]. During vessel sprouting, endothelial cells penetrate the surrounding extracellular matrix, forming columns of migrating and proliferating endothelial cells. These sprouts eventually coalesce to create new vessels with lumens. Activation of endothelial cells prompts the secretion of PDGF-B, which attracts pericytes to these nascent vessels. PDGF-B engages its receptor, PDGFRβ, expressed by pericytes, triggering pericyte proliferation and their recruitment to the newly formed vessels [50, 51]. Subsequently, pericytes contribute to vascular maturation and participate in the remodeling process.

Similar to other cell types, pericytes can undergo apoptosis under specific circumstances. Factors such as oxidative stress, inflammation, growth factor deprivation, and hypoxia can induce pericyte apoptosis [52-54]. In normal physiological contexts, pericyte elimination during tissue remodeling is essential for angiogenesis and vascular maturation. This facilitates endothelial cell sprouting and the formation of new blood vessels, contributing to wound healing and tissue repair [55, 56]. However, pericyte loss due to apoptosis can compromise vascular integrity, disrupt blood flow regulation, and perturb the BBB, culminating in disease progression, as observed in conditions like AD and multiple sclerosis [57]. In our study, we observed a significant increase in the total number of pericytes after 2VO induction. This increase was mainly attributed to the elevated count of detached pericytes. Immunostaining results demonstrated that the detached pericytes were distinct from neurons or glial cells. Notably, some of the attached pericytes appeared to be newly generated cells, implying their proliferation and recruitment to facilitate angiogenesis. Conversely, the majority of detached pericytes exhibited signs of apoptosis, likely induced by CCH. These findings suggest that the presence of detached pericytes might have deleterious effects on the progression of VCI.

Adenosine and its receptors have emerged as promising targets for therapeutic intervention in vascular disorders due to their pivotal role in vascular regulation. The adenosine receptor family consists of four distinct subtypes: A1R, A2AR, A2BR, and A3R, each fulfilling unique physiological functions [24, 58]. A2AR is particularly expressed in myocytes and pericytes, where its activation induces smooth muscle cell relaxation and vasodilation, thereby enhancing local blood flow [59]. This vasodilatory effect is achieved through the activation of potassium channels (KATP) and the inhibition of calcium currents [60, 61]. However, the clinical application of adenosine is hindered by its swift metabolism and clearance from the bloodstream, rendering systemic administration inefficient. In the present study, an innovative strategy was employed, utilizing CGS21680, a selective A2AR agonist. Research has demonstrated that low doses of CGS21680 exhibit neuroprotective effects in rat models of transient cerebral ischemia, marked by a reduction in microgliosis, astrogliosis, and enhanced myelin organization [28]. These beneficial impacts of CGS21680 extend to other conditions, including intracerebral hemorrhage [62], traumatic brain injury [63], and lung inflammation [64]. In our investigation, the administration of CGS21680 yielded several noteworthy outcomes. The treatment led to a pronounced expansion of the capillary lumen at pericyte sites, alongside a significant increase in cerebral blood flow compared to the vehicle-treated group. Moreover, CGS21680 intervention mitigated white matter lesions, evident from the reduction in myelin loss and axonal damage. Furthermore, spatial learning improvements were evident in the Morris Water Maze test following CGS21680 treatment.

In summary, our study elucidates that CCH triggers dysfunction in pericytes, manifesting as perturbations in their quantities and the constriction of capillary lumens encompassing pericyte sites. This aggravates the occurrence of WMLs and cognitive impairment in our rat model of VCI. Encouragingly, our findings indicate that treatment involving CGS21680, a compound inhibiting pericyte constriction, holds the potential to counteract white matter damage, ameliorate cognitive deficits, and confer therapeutic advantages in the context of VCI. In light of these observations, targeting pericytes emerges as a promising therapeutic strategy, wherein the inhibition of their constriction could serve as an effective approach for managing VCI and other related vascular disorders.
